# Digital health innovation to prevent relapse and support recovery in young people with first-episode psychosis: A pilot study of Horyzons-Canada

**DOI:** 10.1038/s41537-023-00352-1

**Published:** 2023-04-07

**Authors:** Shalini Lal, John F. Gleeson, Simon D’Alfonso, Hajin Lee, Geraldine Etienne, Ridha Joober, Martin Lepage, Mario Alvarez-Jimenez

**Affiliations:** 1 School of Rehabilitation, Faculty of Medicine, University of Montréal, Montréal, QC Canada; 2 Youth Mental Health and Technology Lab, Health Innovation and Evaluation Hub, University of Montréal Hospital Research Centre, Montréal, QC Canada; 3 Prevention and Early Intervention Program for Psychosis, Douglas Mental Health University Institute, Montréal, QC Canada; 4 Healthy Brain and Mind Research Centre and School of Behavioural and Health Sciences, Australian Catholic University, Fitzroy, VIC Australia; 5 School of Computing and Information Systems, University of Melbourne, Parkville, VIC Australia; 6 Department of Psychiatry, McGill University, Montréal, QC Canada; 7 Centre for Youth Mental Health, University of Melbourne, Parkville, VIC Australia; 8 Orygen, Parkville, VIC Australia

**Keywords:** Psychosis, Schizophrenia

## Abstract

Digital health innovations may help to improve access to psychosocial therapy and peer support; however, the existence of evidence-based digital health interventions for individuals recovering from a first-episode psychosis (FEP) remains limited. This study aims to investigate the feasibility, acceptability, safety, and pre-post outcomes of Horyzons-Canada (HoryzonsCa), a Canadian adaptation of a digital mental health intervention consisting of psychosocial interventions, online social networking, and clinical and peer support moderation. Using a convergent mixed-methods research design, we recruited participants from a specialized early intervention clinic for FEP in Montreal, Canada. Twenty-three participants (mean age = 26.8) completed baseline assessments, and 20 completed follow-up assessments after 8 weeks of intervention access. Most participants provided positive feedback on general experience (85%, 17/20) and the utility of Horyzons for identifying their strengths (70%, 14/20). Almost all perceived the platform as easy to use (95%, 19/20) and felt safe using it (90%, 18/20). There were no adverse events related to the intervention. Participants used HoryzonsCa to learn about their illness and how to get better (65%, 13/20), receive support (60%, 12/20), and access social networking (35%, 7/20) and peer support (30%, 6/20). Regarding adoption, 65% (13/20) logged in at least 4 times over 8 weeks. There was a nonsignificant increase in social functioning and no deterioration on the Clinical Global Impression Scale. Overall, HoryzonsCa was feasible to implement and perceived as safe and acceptable. More research is needed with larger sample sizes and using in-depth qualitative methods to better understand the implementation and impact of HoryzonsCa.

## Introduction

Evidence supports the effectiveness of psychosocial interventions (e.g., cognitive behavioral therapy, psychoeducation, peer support) provided to young people experiencing first-episode psychosis (FEP)^1^. Yet, universal and sustained access to these interventions continues to be a challenge^1–5^. This is especially true in a paradigm that has predominantly relied on traditional models of delivering services in person and individually. In response, there has been increased attention on the potential of digital interventions (beyond videoconferencing) to support clinical and social outcomes in individuals living with schizophrenia spectrum and other psychotic disorders, including FEP^6–8^.

One such intervention is Horyzons, a digital health intervention originally designed to support recovery and transition from specialized services in young adults with an FEP^9^. Horyzons is delivered through a web-based application that consists of interactive, evidence-based-strengths-focused psychosocial interventions, online social networking, and clinical and peer support moderation^9^. Horyzons was originally developed and pilot-tested in Australia^9^, followed by a randomized controlled trial (18-month, parallel-group, single-blind)^10^. Recently, international interest in Horyzons has led researchers to evaluate this intervention in the U.S.^11^, the Netherlands^12^, and Canada^13^. Horyzons has also been adapted for young people at ultra-high risk for psychosis^14^, and populations with other mental illnesses such as depression^15,16^.

In Canada, an adaptation study on Horyzons was conducted^13,17^, followed by a pilot study. In this manuscript, we report on the pilot study results. The purpose was to evaluate the feasibility of implementing and evaluating the adapted version of Horyzons in a Canadian context (HoryzonsCa), and its acceptability, safety, and pre-post outcomes using an uncontrolled single-group, pre-post (8 weeks), mixed methods (QUAL-QUAN convergent) design. The feasibility of conducting HoryzonsCa was informed by data on recruitment rates and the appropriateness of eligibility criteria. The primary hypothesis was that the intervention will be considered acceptable, defined as at least 70% of participants providing positive reports on general experience of the platform; 60% providing positive reports on perceived usefulness (helpfulness) and ease of use; and 60% logging onto the site at least 4 times over 8 weeks. The login cut-off was informed based on preliminary international data with the intervention. For example, minimum exposure to the intervention was defined as at least one login per week in pilot research with 12 weeks follow-up^11^, and the original pilot study on Horyzons showed that 70% of participants had more than 6 logins per month^9^. However, more recently, a randomized controlled trial (RCT) study on Horyzons set the minimum exposure to the intervention as a rate of 1 login/8 weeks^10^ within the context of a longer follow-up of 18 months. Thus, as part of our protocol, we hypothesized that at least 60% of participants will have 4 logins or more over the 8 weeks follow-up. The secondary hypotheses were: (1) HoryzonsCa will be safe, defined as no adverse events, reports, or incidents (e.g., hospitalization, suicidal ideation, or disclosure to treatment team regarding harm) related to the use of the platform over 8 weeks, and at least 70% of participants reporting that they agree or strongly agree with the perceived safety and confidentiality on the platform; and (2) participants will show social functioning improvements and either improvement or no deterioration on the Clinical Global Impression Scale over 8 weeks (primary foci for assessing pre-post outcomes). Extensive details on Horyzons, previous research on Horyzons, and the research protocol methods used in this study have been previously published^18^. Here we attend to the details of the results and then provide an overview of the methods.

## Results

### Baseline sociodemographic and clinical characteristics

As part of the baseline assessment, 23 participants reported their sociodemographic characteristics. The mean age of the sample was 26.8 years (SD = 5.3; range = 18–36), of which 52% (12/23) identified as female, 57% (13/23) used mental health services less than or equal to 1 year, and 96% (22/23) had at least a high school diploma. At baseline, participants showed some difficulties in social, occupational, or school functioning but were generally functioning well, given their high social functioning scores (SOFAS, PSP = 65). See Table 1 for a summary of the participant sociodemographic characteristics. Further details regarding the baseline clinical characteristics of participants are provided in the results on pre-post outcomes (see Table 5).

**Table 1:** Baseline sociodemographic characteristics of FEP patients (*N* = 23).

Characteristic	Mean ± SD or *n* (%)
Age (years)	26.8 ± 5.3 (18–36)
Sex^a^	
Female	12 (52)
Male	10 (43)
Other	1 (4)
Length of service use^b^	
0–12 months	13 (57)
More than 1 year	8 (35)
Education	
Less than high school	1 (4)
High school	8 (35)
College diploma/certificate	12 (52)
Bachelor’s degree	2 (9)
Ethnicity^a,c^	
White	12 (52)
Black	4 (17)
Latin American	3 (13)
Other (“Indigenous”; “Chinese”; “South Asian”; “Arab”)	5 (22)
Vocational status^c^	
Working [part-time: 3, full-time: 6]	9 (39)
School [part-time: 3, full-time: 5]	8 (35)
Other (“unemployment”; “sick leave”; “soon school part-time”; “welfare”; “searching for work”)	10 (43)
Living situation^c^	
Alone	5 (22)
Parents/siblings	14 (61)
Partner	2 (9)
Other (“friend/roommate”; “residence/group home”; “cousin”)	3 (13)
Marital status	
Single	15 (65)
In a relationship	7 (30)
Separated/divorced	1 (4)
Income	
Under $14,999/year	16 (70)
$15,000–$29,000/year	2 (9)
$30,000–$49,999/year	2 (9)
$50,000 and over/year	1 (4)
Prefer not to disclose	2 (9)

^a^Self-identified.

^b^Missing data on length of service use in 2 cases (9%).

^c^More than one answer possible.

### Technology access, use, and competency

Participants were asked about their technology access, use, and competency level at baseline and 8 weeks follow-up. The results from the mixed effects logistic regression and McNemar’s test indicated that overall, there were no differences in general access and use of technology, and frequency (except the use of email to communicate with others) and competency of technology use between baseline and follow-up. The majority of participants had access to a smartphone, a data plan, a computer at home, and the internet. Most reported using the internet to search for mental health support once per week or less, with Google being the most common site used for these searches. The majority indicated they use social media, text, and email to communicate with others. For participants who indicated they did not use social media, some of the reasons were: they felt it was bad for mental health, they used it a lot in the past but not anymore, they only used it for school, and they just did not like it. Reasons for not using email included: that it is “old fashioned,” they use other methods to communicate (i.e., phone, app, text), they have no need to use it, they use it only for professional communication, and that it is too long or there is too much spam. Competency level with various forms of technology was reported high, with the majority of participants feeling very competent using a computer, navigating websites, and using social media. Most participants also reported feeling very competent or somewhat competent searching the internet for mental health information, services, and supports. See Supplementary Table 1 for additional details.

### Feasibility

**Fig. 1:**
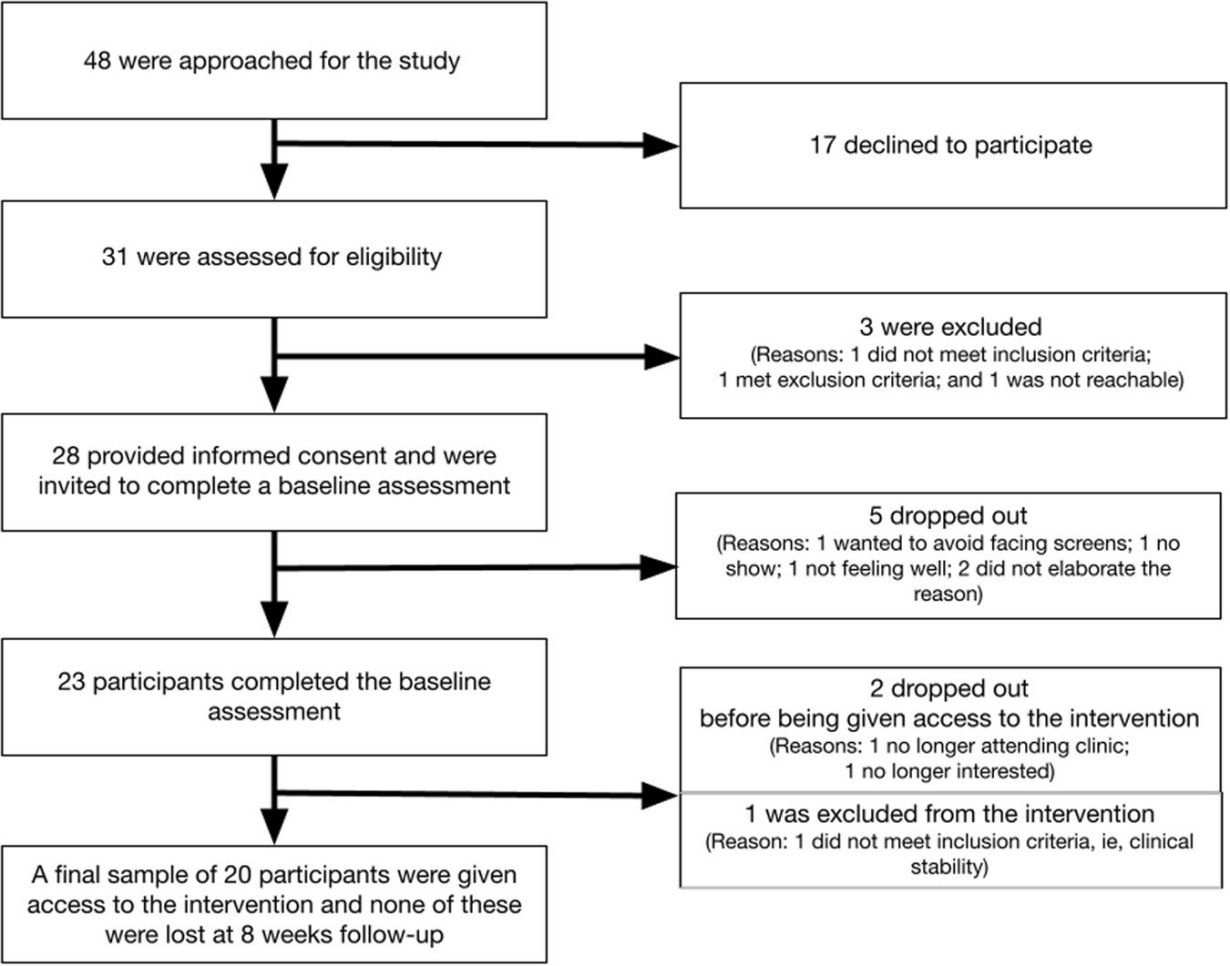
Recruitment process. Recruitment was initiated on May 10, 2018 and completed over approximately 9 months, with a monthly recruitment rate of approximately 3 participants.

Details on the recruitment process and outcomes are provided in our protocol paper^18^ and summarized in Fig. 1. Among the 28 individuals that provided informed consent, 23 completed a baseline assessment. Out of 23, one was deemed ineligible after the baseline assessment due to clinical instability, and two dropped out before being given access to the intervention. Among the 20 participants who were given access to the intervention, none were lost at 8 weeks follow-up. To assess pre-post outcomes, the analyses included 23 participants who completed the baseline assessment and 20 participants who completed the follow-up over 8 weeks. Recruitment was initiated on May 10, 2018, and data collection occurred between August 16, 2018, to April 29, 2019. Recruitment was completed over approximately 9 months, with a recruitment rate of approximately 3 participants per month.

### Acceptability and safety

Acceptability of HoryzonsCa was determined based on perceptions regarding general experience, usefulness, ease of use, and actual adoption (i.e., website usage). Safety was determined based on the perceived safety and confidentiality of HoryzonsCa and adverse events, reports, or incidents related to the intervention. Responses to closed-ended questions regarding the acceptability and safety of HoryzonsCa are detailed in Table 2. Short responses to open-ended questions are detailed in Supplementary Table 2.

**Table 2:** Quantitative feedback from the HC-AUSI-Q (*N*_total_ = 20).

Questions regarding the acceptability and safety of HoryzonsCa	*n*	%
*General experience*		
I had a positive experience on Horyzons-Canada		
Strongly agree/agree	17	85
Neutral	3	15
Strongly disagree/disagree	0	0
I would recommend the use of Horyzons-Canada to other people		
Strongly agree/agree	18	90
Neutral	2	10
Strongly disagree/disagree	0	0
*Perceived usefulness*		
Horyzons was useful to identify my strengths		
Strongly agree/agree	14	70
Neutral	3	15
Strongly disagree/disagree	3	15
The moderators and super-users provided helpful feedback		
Strongly agree/agree	16	80
Neutral	4	20
Strongly disagree/disagree	0	0
Horyzons-Canada helped me to get in touch with other people and feel more socially connected		
Strongly agree/agree	7	35
Neutral	7	35
Strongly disagree/disagree	6	30
Other Horyzons-Canada users helped my recovery on Horyzons-Canada		
Strongly agree/agree	4	20
Neutral	7	35
Strongly disagree/disagree	9	45
*Ease of use*		
Overall, the platform is easy to use		
Strongly agree/agree	19	95
Neutral	1	5
Strongly disagree/disagree	0	0
The information on Horyzons-Canada was easy to understand		
Strongly agree/agree	19	95
Neutral	0	0
Strongly disagree/disagree	1	5
*Safety*		
I felt safe on Horyzons-Canada		
Strongly agree/agree	18	90
Neutral	1	5
Strongly disagree/disagree	1	5
I felt like the information shared on Horyzons-Canada was confidential		
Strongly agree/agree	18	90
Neutral	1	5
Strongly disagree/disagree	1	5

*HC-AUSI-Q* Horyzons-Canada Acceptability, Usability, Safety, and Impact Questionnaire.

The majority agreed or strongly agreed that they had a positive experience using HoryzonsCa (85%, 17/20) and would recommend it to other people (90%, 18/20). The majority agreed or strongly agreed that it was useful to identify their strengths (70%, 14/20) and that the moderators provided helpful feedback (80%, 16/20). The peer-to-peer aspects of HoryzonsCa were perceived as follows: 35% (7/20) agreed and 35% (7/20) were neutral with the statement that HoryzonsCa helped them get in touch with other people and feel socially connected; and 20% (4/20) agreed and 35% (7/20) were neutral with the statement that other users helped their recovery on the platform. Eighteen participants provided additional comments regarding the extent to which they perceived other users as being helpful on the platform, which we categorized as follows: 56% (10/18) described positive interactions with moderators, but neutral or less positive interactions with other users; some participants (44%, 8/18) also highlighted aspects they perceived as being unhelpful, such as low user activity and interactions on the platform.

Almost all participants agreed or strongly agreed that the platform was easy to use (95%, 19/20) and the information on HoryzonsCa was easy to understand (95%, 19/20). Seventeen participants provided additional comments regarding whether the information on Horyzons-Canada was easy to understand, of which 59% (10/17) highlighted the clarity of content (e.g., simple text) and 35% (6/17) expressed a liking for the format of content (e.g., comic scripts).

In line with our secondary hypothesis about safety, there were no adverse events, reports, or incidents (e.g., hospitalizations, suicidal ideations, disclosure to the treatment team regarding harm) related to the use of HoryzonsCa from baseline to 8 weeks. The majority felt safe on Horyzons Ca (90%, 18/20) and that the information remained confidential (90%, 18/20). Overall, participants highlighted positive perceptions regarding the security of the system and user safety. Regarding user safety, some noted trust in the clinical moderators’ credibility, understanding, and professionalism as positive components. Comments regarding system safety pertained to the password-protected feature of the platform, no advertisements, anonymity (i.e., not knowing other participants personally), and privacy of the posts (i.e., knowledge the posts are not being used elsewhere).

In terms of adoption, on average, participants logged into the platform 7.10 times over the 8-week follow-up period (*SD* = 7.30, median = 5.00, range = 0–30), and most participants logged into the platform 4 times or more over 8 weeks (65%, 13/20). We defined minimal platform usage as an average of at least one login per 2 weeks (4 total logins). Active participants (*n* = 13) met this standard, whereas inactive participants (*n* = 7) did not reach minimum usage. Additional HoryzonsCa usage information is provided in Table 3.

**Table 3:** HoryzonsCa usage information over the 8 weeks pilot study (*N*_total_ = 20)^a^.

HoryzonsCa component	All^b^, *n* = 20					Active participants^b,c^, *n* = 13				
	Mean	SD	Range	Median	Q1–Q3	Mean	SD	Range	Median	Q1–Q3
Logins^d^	7.10	7.30	0–30	5	2–10	10.23	7.34	4–30	9	5–13
Newsfeed posts^e^	0.20	0.52	0–2	0	0–0	0.31	0.63	0–2	0	0–0.5
Newsfeed comments^f^	1.20	2.31	0–10	0.5	0–1	1.85	2.67	0–10	1	0.5–2
Pathways completed^g^	0.15	0.49	0–2	0	0–0	0.23	0.60	0–2	0	0–0
Steps completed^h^	3.15	4.83	0–18	1.5	0–4	4.69	5.42	0–18	3	1.5–5.5
Actions completed^i^	2.65	5.74	0–24	0	0–2.5	3.77	6.91	0–24	1	0–6.5
Talk-it-outs^j^	0.20	0.62	0–2	0	0–0	0.31	0.75	0–2	0	0–0

^a^Covers the period from July 24, 2018, to April 24, 2019; participants were recruited into the system from July 24, 2018, to February 27, 2019.

^b^Data are mean, standard deviation, range, and median (25th quartile–75th quartile, Q1–Q3).

^c^Participants who logged into the platform 4 times or more. We chose the cutoff value of 4 based on our definition of minimum platform usage as an average of at least one login per 2 weeks (4 total logins) during the period of an 8-week intervention.

^d^Number of logins over 8 weeks.

^e^Number of newsfeed posts made by participants in the peer-to-peer web-based social networking (the Café).

^f^Number of newsfeed comments made by participants in the peer-to-peer web-based social networking (the Café).

^g^Number of Pathways that participants completed as part of engagement with therapeutic content.

^h^Number of Steps (each “Pathway” comprised of a series of related “Steps”) that participants completed as part of engagement with therapeutic content.

^i^Number of Actions (activities designed to reinforce strengths or practice new skills) that participants completed as part of engagement with therapeutic content.

^j^Number of Talk-it-outs posts and comments in which participants discuss specific issues (e.g., handling setbacks) and receive support or suggestions.

### Engagement and motivation for use

When asked about the reasons why they chose to use HoryzonsCa, most participants indicated learning about their illness experience and knowledge on how to get better (65%, 13/20), and receiving support from mental health service providers (60%, 12/20). In terms of their perceptions of how long a young person should engage with HoryzonsCa, more than half (55%, 11/20) indicated between 1 and 12 months, and some (25%, 5/20) indicated even longer (13–24 months). Further details are provided in Table 4.

**Table 4:** Quantitative feedback from the HC-AUSI-Q (*N*_total_ = 20).

Questions regarding engagement and motivation for the use of HoryzonsCa	*n*	%
*When I used Horyzons-Canada it was mostly for [check all that apply]*^a^		
Learning what happened to me and how to get better		
Yes	13	65
No	6	30
Receiving support from mental health service providers		
Yes	12	60
No	7	35
Giving support to others		
Yes	9	45
No	10	50
Social networking (meeting other youth who have gone through similar experiences)		
Yes	7	35
No	12	60
Receiving support from other youth		
Yes	6	30
No	13	65
Other purpose (“knowledge”; “learning new things”; “curiosity”; “journal where to write opinions, where questions like how do you feel today?”; “I guess it was just for the audio”; “see what they say about mental health in general”)		
Yes	6	30
No	13	65
*How long do you think a young person should use Horyzons-Canada*^a^		
1–3 months	4	20
4–6 months	4	20
7–12 months	3	15
13–24 months or more	5	25
Other (“neutral, customize duration—ask participants”; “it depends on the age”; “depends on the person, e.g., people with less experience”)	3	15

*HC-AUSI-Q* Horyzons-Canada Acceptability, Usability, Safety, and Impact Questionnaire.

^a^Missing data on the question in 1 case (5%).

We asked participants to comment on barriers and strategies that would foster a young person’s engagement on HoryzonsCa. Obstacles were categorized into those related to the system or user. In terms of the system, the most common obstacles pertained to technical (e.g., having to remember another password) or content (e.g., childish cartoons, the amount of reading, lack of video content). Common user obstacles were lack of time or willingness to use the platform, or lack of comfort with sharing personal feelings and experiences, particularly with those who are not well-known to users.

In terms of perceived facilitators to engaging with HoryzonsCa, the most common responses were improving system features (e.g., tracking follow-ups with users, and creating a mobile app). Another common response pertained to including more engaging content (e.g., video games or videos). Some participants expressed improving user interactions on the platform (42%, 8/19), and others highlighted the importance of promoting the platform more broadly using various methods such as social media (32%, 6/19). Participants also provided suggestions to introduce youth to the platform for the first time, including using videos and/or social media and demonstrations by professionals or peers (see Supplementary Table 2 for details).

### Pre-post outcomes

As illustrated in Table 5, we evaluated participants’ improvements in outcomes from baseline to 8 weeks of follow-up using linear mixed models. As for our primary foci for assessing pre-post outcomes, participants showed a nonsignificant change in social functioning (SOFAS: *b* = 4.24, 95% confidence interval (CI) = [−4.78, 13.26], *p* = 0.35; PSP: *b* = 6.50, 95% CI = [−3.55, 16.56], *p* = 0.20) and no deterioration on the Clinical Global Impression Scale (*b* = 0.14, 95% CI = [−0.79, 1.07], *p* = 0.76), from baseline to follow-up. In terms of secondary outcomes, participants’ perceived social support, self-esteem, perceived strengths, and symptoms demonstrated no significant changes from baseline to follow-up. Although it is not significant, there was an upward trend in social support (*b* = 0.63, 95% CI = [−0.31, 1.58], *p* = 0.18), self-esteem (*b* = 11.54, 95% CI = [−18.97, 42.05], *p* = 0.45), strengths use (SUS: *b* = 2.62, 95% CI = [−8.07, 13.32], *p* = 0.62), and there was a downward trend in the presence of symptoms, including psychiatric symptoms (*b* = −4.60, 95% CI = [−10.18, 0.98], *p* = 0.10), negative symptoms (*b* = −1.56, 95% CI = [−3.85, 0.73], *p* = 0.18), positive symptoms (*b* = −1.17, 95% CI = [−2.92, 0.59], *p* = 0.19), and depressive symptoms (*b* = −0.76, 95% CI = [−3.27, 1.76], *p* = 0.55). Additionally, an exploratory analysis showed significant associations between steps taken and changes in pre-post CGI scores (*r* = −0.57, *p* = 0.03), and between actions completed and changes in pre-post CGI scores (*r* = −0.59, *p* = 0.03). See Supplementary Table 3 for details.

**Table 5:** Means, standard deviations, and linear mixed models examining time as a predictor of social functioning and clinical measures.

Measure	Baseline (*N* = 23)		8 Weeks follow-up (*N* = 20)		Estimate (95% CI)	*p* value
	Mean	SD	Mean	SD		
SOFAS^a^	65.39	13.91	69.63	14.96	4.24 (−4.78, 13.26)	0.35
PSP^b^	65.22	14.48	71.72	17.35	6.50 (−3.55, 16.56)	0.20
CGI^c^	2.52	1.44	2.67	1.23	0.14 (−0.79, 1.07)	0.76
Global SANS^d^	7.09	3.74	5.53	3.55	−1.56 (−3.85, 0.73)	0.18
BPRS^e^	39.65	8.77	35.05	9.08	−4.60 (−10.18, 0.98)	0.10
MSPSS	4.09	1.66	4.73	1.38	0.63 (−0.31, 1.58)	0.18
CDS^f^	2.91	3.36	2.16	4.69	−0.76 (−3.27, 1.76)	0.55
Global SAPS^g^	3.70	2.88	2.53	2.70	−1.17 (−2.92, 0.59)	0.19
SES	37.26	51.02	48.80	47.48	11.54 (−18.97, 42.05)	0.45
SKS	28.52	8.48	27.85	11.80	−0.67 (−6.94, 5.60)	0.83
SUS	61.83	14.87	64.45	19.78	2.62 (−8.07, 13.32)	0.62

*CI* confidence interval, *SOFAS* Social and Occupational Functioning Assessment Scale, *PSP* Personal and Social Performance Scale, *CGI* Clinical Global Impression, *SANS* Scale for the Assessment of Negative Symptoms, *BPRS* Brief Psychiatric Rating Scale, *MSPSS* Multidimensional Scale of Perceived Social Support, *CDS* Calgary Depression Scale, *SAPS* Scale for the Assessment of Positive Symptoms, *SES* Self-Esteem Rating Scale, *SKS* Strengths Knowledge Scale, *SUS* Strengths Use Scale.

^a^The SOFAS was answered by 19 participants in the follow-up. The range of SOFAS scores was 1 to 100, and higher scores indicate better functioning in social, occupational, or school functioning.

^b^The PSP was answered by 18 participants in the follow-up. The range of PSP scores was 1 to 100, and higher scores indicate better functioning in all four main areas: socially useful activities (including work and study), personal and social relationships, self-care, and disturbing and aggressive behaviors.

^c^The CGI was answered by 21 participants in the baseline and 15 participants in the follow-up. The range of CGI scores was 1 [very improved—nearly all better good level of functioning; minimal symptoms; represents a very substantial change] to 7 [very much worse—severe exacerbation of symptoms and loss of functioning].

^d^The Global SANS was answered by 19 participants in the follow-up. The global scores of SANS were calculated based on the sum of global rating items 1 to 4, excluding the global rating item of attention^43,44^.

^e^The BPRS was answered by 19 participants in the follow-up. The total scores of BPRS were calculated based on items 1 to 24, excluding the items P11 and P12.

^f^The CDS was answered by 19 participants in the follow-up.

^g^The Global SAPS was answered by 19 participants in the follow-up. The global scores of SAPS were calculated based on the sum of the global items^43^.

## Discussion

### Principal findings

The preliminary results of this study support the feasibility and acceptability of delivering HoryzonsCa to young adults recovering from FEP. The eligibility criteria were appropriate (with only one participant not meeting eligibility criteria out of the 28 participants who provided informed consent), and the study’s reasonable patient recruitment rate for a clinical population known to be difficult to engage (approximately 3 participants per month in a single center) in the context of an intervention study. Implementation challenges included: recruiting peer support moderators and clinical moderators, and providing ongoing training and supervision to individuals that were moderating online for the first time.

Nonetheless, as predicted, HoryzonsCa was accepted by and was found to be safe for FEP patients. The majority provided positive feedback on general experience, perceived usefulness (helpfulness), ease of use, and safety of the platform; the adoption rate was high, with 65% logging onto the platform at least 4 times over 8 weeks, and no adverse events or incidents occurred with the intervention. Overall, these results are in line with the findings from the first pilot study on Horyzons, conducted in Australia with 20 young adults, that reported 70% logging onto the platform at least 6 times over 4 weeks^9^. However, this study is distinguished from previous research on Horyzons, as it is the first to report on a systematic adaptation process^13,17^ that considers geographical, cultural, and healthcare contexts. This is relevant given that evidence suggests that adaptation of a psychosocial intervention (delivered in-person or web-based) (e.g., considering language, culture, and context) can contribute positively to its adoption and effectiveness^17,19,20^.

While Australia and Canada have many similarities, contextual factors such as bilingualism in health care settings (which also extends to eHealth), differences in the use of English terms and colloquialisms, and systems-level differences in community resources and how mental health services are organized and delivered (e.g., lack of formally trained peer support workers, clinicians trained in the delivery of digital health interventions, technology infrastructure) are important factors to consider for the feasibility of transporting a digital health intervention from one context to another. Indeed, based on our phase 1 research, we adapted several aspects of the intervention, detailed in our previous publication, including but not exclusive to terms and colloquialisms, safety and moderation protocols, need help resources, terms of use, list of trigger (risk) words automatically flagged by the system, change to content and resources pertaining to employment/studying/volunteering to be in alignment with Canadian norms^13^. The acceptability of the intervention may be partially attributed to these adaptation processes, as suggested by previous literature^13,19–21^.

In terms of perceived usefulness (helpfulness), the majority of the participants perceived the platform and moderators to be helpful. However, only approximately a third found that the intervention helped them feel socially connected or felt helped by others in terms of their recovery. This may partially be attributed to limited usage of the social networking features (e.g., number of newsfeed posts, talk-it-outs), which in turn may be due to the gradual recruitment of participants (i.e., participants not having access to the intervention at the same time as many others, limiting opportunities to use the social networking features). It is also possible that a lack of familiarity with other users contributed to participants’ reticence to use the social networking features, as mentioned by some participants in the open-ended responses. In previous qualitative research, authors reported other factors contributing to barriers to the use of Horyzons, such as social anxiety, paranoia, internalized stigma, lack of autonomy, and uncertainties regarding social protocols^22^.

With respect to pre-post outcomes in social functioning and clinical measures, there was no observed deterioration on the Clinical Global Impression Scale (that was used to assess participants’ global improvement and severity of illness) at follow-up. Contrary to findings from a recent RCT conducted on Horyzons in Australia, our study did not show significant improvements in social functioning. This may be partially attributed to factors such as good levels of social functioning at baseline (that may be related to recruitment bias), hence limited room for improvement^10^; the short duration of the pilot testing (i.e., 8 weeks); the limited number of participants on the platform at any given time (the highest number of participants on the platform was 10; these participants were within varying stages of their time on the platform, i.e., either at the initial-point, mid-point or end-point of their 8 weeks follow-up); the lack of tailored interventions on vocational, educational, or related functional skills; the limited sample size; and instrument sensitivity. These aspects may be given more attention in a larger trial, with specialized moderation (e.g., from a vocational specialist or occupational therapist).

Although the field of digital health innovation is rapidly growing^23,24^, the limited evidence on the implementation of digital mental health interventions for individuals with FEP may limit the scope of our interpretations. Factors that contribute to this limited evidence base include: the rapidity of technological evolution; the complexity that digital mental health interventions seek to address; and the limitations of traditional health research methods such as randomized controlled trials (developed for evaluating pharmaceuticals) for the evaluation of digital mental health interventions^24^. As such, newer, agile approaches to the evaluation of digital mental health interventions need to be considered, including those that allow for quality improvement of the intervention and the technology during a trial^24^. Aligned with these considerations, our next step will be to scale up the evaluation of the acceptability, safety, and pre-post outcomes of HoryzonsCa to a larger implementation study currently underway. This larger study aims to deliver the intervention to a sample of 100 to 150 patients with schizophrenia spectrum and other psychotic disorders across the trajectory of illness aged 18 to 50 over a longer duration (12 weeks), recruited from two sites, implemented in a bilingual health service context (English and French), and supported through a more comprehensive moderator training and implementation strategy. The evaluation will allow for learning and optimization of the intervention and its related technology during the trial to increase utility of the evidence that is generated and to ensure currency of the findings with the technological environment at the time of study completion.

### Limitations

Limitations include a small sample size and a single group, pre-post design. Due to this small sample size (*N*_baseline_ = 23, *N*_follow-up_ = 20), the short duration of follow-up (i.e., 8 weeks), and a lack of a control group, our analysis may lack statistical power. Due to this study’s exploratory aims and its scope being a pilot, we conducted several exploratory tests with no adjustment applied for multiple comparisons (including Bonferroni corrections). Thus, our preliminary findings should be interpreted with caution. This study focused on total logins over the 8 weeks follow-up period. Thus, no distinction was made with those logging more frequently at different stages of the follow-up. However, questions regarding minimal exposure, categorizing of active vs. inactive, and time of engagement in the intervention are areas to consider for future research. In addition, considering the clinical characteristics of the sample (*N* = 23) at baseline, participants were functioning relatively well before the intervention limiting the possibility of identifying differences in pre-post outcomes; this may reflect a recruitment bias towards the identification of higher-functioning FEP patients and a participation bias in high-functioning patients that are more likely to accept study participation. To address some of these limitations, future studies should compare the sociodemographic characteristics of study participants to individuals who were not invited to participate or dropped out. Moreover, future studies should have a larger sample size to establish treatment effects, and consider other outcome measures, such as relapse. Finally, research using qualitative methods may help unpack the intervention’s impact on recovery, the various obstacles to engaging with the platform, and strategies to overcome them.

## Methods

### Study design

This cohort study is implemented in a single center and applies a pre-post mixed-methods (qualitative-quantitative convergent) design. The study involves recruiting a target sample of 20 participants from a specialized early intervention program for FEP located in Montreal, Canada, and providing them with access to HoryzonsCa for 8 weeks with pre-post follow-up assessments. Horyzons was originally informed by the Moderated Online Social Therapy (MOST) model, combining moderated social networking and therapy components into a fully integrated system^25^. Details of our data collection methods and measures are available in our published protocol^18^. The study was also registered in the International Standard Randomized Controlled Trial Number (ISRCTN) online public registry on November 1, 2018 (trial number: ISRCTN43182105; https://www.isrctn.com/ISRCTN43182105). The study was approved by the Research Ethics Board of the Centre intégré universitaire de santé et de services sociaux de l’Ouest-de-l’Île-de-Montréal on April 11, 2018 (#IUSMD 17–54). All participants provided written informed consent.

### Target population

Participants were recruited from the Prevention and Early Intervention Program for Psychosis (PEPP)–Montreal located at the Douglas Mental Health University Institute, affiliated with McGill University, in Montreal, Quebec. PEPP targets individuals with FEP, specifically, serving 14- to 35-year-olds with a diagnosis of affective or non-affective psychosis who have had no more than 1 month’s previous antipsychotic treatment; without organic brain damage, a pervasive developmental disorder, an IQ below 70, or epilepsy; and do not have substance-induced psychosis. A comorbid diagnosis of substance abuse is not an exclusion criterion for access to the program^26^. *Participant inclusion criteria* for this study were (1) diagnosis of a psychotic disorder (including affective or non-affective psychoses); (2) receiving specialized early intervention (EI) services for an FEP at PEPP, which provides specialized, phase-specific, developmentally informed, comprehensive treatment for the first 2 years after diagnosis^26^, following consensus guidelines^27^; (3) considered symptomatically stable and capable of interacting on the online platform and participating in focus groups and semi-structured interviews, as judged by their primary clinicians (i.e., psychiatrist, case manager); (4) 18 years of age or older; (5) at low or at most moderate severity score (4 or below) on the suicidality item of the Brief Psychiatric Rating Scale, version 4^28^, for the month preceding study entry. Given the nature of this intervention study as a pilot for feasibility, especially for the moderators, we set this suicidality score criterion to minimize risk for the need for urgent intervention regarding the posting of comments about suicidality posted on the social networking or to the moderation team (given that the platform is not monitored 24/7) and to reduce potential anxiety or distress in other participants from overexposure to suicide-focused content; and (6) able to nominate an emergency contact. *Participant exclusion criteria* included (1) intellectual disability; (2) hospitalized at the time of recruitment; (3) unable to speak or read English; (4) diagnosis of antisocial personality disorder and/or borderline personality disorder; and, (5) in the acute phase of mania or psychosis to the extent that their mental status may soon require hospitalization, or would impede the participant’s ability to provide informed consent or to participate in interviews.

### Intervention

Horyzons is an online intervention that was originally designed to sustain the treatment benefits of early intervention for psychosis and to promote long-term social functioning^9^. However, within the context of this pilot study in Canada, based on feedback from clinicians and patients from our phase 1 adaptation study, we evaluated HoryzonsCa as an intervention delivered concurrently with early intervention services. This intervention delivers evidence- and strengths-based targeted psychosocial interventions and is enhanced by a moderated online social networking environment. Youth diagnosed with FEP are guided through interactive activities to identify, discuss, and develop key personal strengths to address relapse risk factors and psychological well-being. Specifically, Horyzons consists of *interactive psychosocial interventions* that are informed by evidence-based psychosocial interventions targeting key risk factors and salient domains in the early recovery process (including psychoeducation, vocational recovery, early warning signs of relapse, depression, social anxiety, and personal strengths); *peer-to-peer web-based social networking* that includes a web feed (or news feed) where youth with FEP and moderators can post comments and information, upload pictures and videos, and *like* different types of content; and *moderation* that is conducted by clinicians and peer support workers. Clinician moderators provide guidance, monitor clinical status, and ensure safety of the social networking environment. Peer support moderators are young adults with lived experience who have received peer support training and who have been stable and in remission for a minimum of 2 years. Their role includes assisting with orientation to the Horyzons intervention platform, providing support, and fostering engagement. The intervention platform includes a comprehensive safety protocol following best practices in internet research involving vulnerable people^29^, which considers 3 levels of security (i.e., online safety, clinical safety, and system security). Additional details on the design, development, and Canadian adaptation of Horyzons have been previously published^9,10,18^.

### Measures

*Feasibility* was assessed by collecting data on recruitment rates and appropriateness of eligibility criteria to inform the feasibility and design for a future larger implementation study.

*Acceptability and perceived safety* of HoryzonsCa were assessed through the Horyzons-Canada Acceptability, Usability, Safety, and Impact Questionnaire (HC-AUSI-Q)^18^ and website usage analytics (e.g., number of logins, number of steps taken). The interviewer-administered HC-AUSI-Q consists of a questionnaire and a semi-structured interview that includes 16 closed and 10 open-ended questions on perceived ease of use, perceived usefulness, enjoyment, and safety. *Perceived safety* was measured using two specific questions (i.e., “I felt safe on Horyzons-Canada,” “I felt like the information shared on Horyzons-Canada was confidential”) in the HC-AUSI-Q. In addition, any adverse events, reports, or incidents (e.g., hospitalization, suicidal ideation, disclosure to treatment team regarding harm) regarding the use of the online system were carefully monitored and quantified over the study duration.

*Pre-post outcomes* of the intervention were assessed using social functioning and clinical measures. In terms of *primary outcome measures*, social functioning was measured using the interviewer-administered Social and Occupational Functioning Assessment Scale^30^ and the interviewer-administered Personal and Social Performance Scale^31^. The following *secondary outcome measures* were also used: the Clinical Global Impression Scale^32^ to assess global improvement and therapeutic response; the Multidimensional Scale of Perceived Social Support^33^ to assess social support; the Self-Esteem Rating Scale^34^ to assess self-esteem; the Strengths Knowledge Scale^35^ and the Strengths Use Scale^35,36^ to assess perceived strengths; and the Scale for the Assessment of Positive Symptoms^37^, the Scale for the Assessment of Negative Symptoms^38^, the Brief Psychiatric Rating Scale^28^, and the Calgary Depression Scale^39^ to assess mental health symptoms. Additional details on these measures are provided in our protocol^18^.

In addition to open-ended questions through the HC-AUSI-Q, we also collected data through several focus groups. This aspect of the study generated a rich verbatim data set, and will therefore be reported in a subsequent paper to provide the opportunity to delve into detail regarding the experiences and perceptions of participants using HoryzonsCa.

### Procedures

We collected quantitative and qualitative data through interview-based psychometric measures and self-reports. After providing written informed consent, a research assistant (independent from intervention delivery) administered the *HoryzonsCa Initial Interviews and Orientation Meeting* and the *HoryzonsCa Exit Interview*. The initial interview consisted of completing a self-reported sociodemographic questionnaire, the Technology Access, Use, and Competency Questionnaire (TAUC-Q), and a combination of self-reported and interviewer-administered clinical measures (for social functioning, global improvement and therapeutic response, social support, self-esteem, perceived strengths, and symptoms). The TAUC-Q and the clinical measures were also administered during the exit interview at the 8 weeks follow-up along with the interviewer-administered HC-AUSI-Q.

### Statistical analyses

As described in our protocol^18^, following the convergent mixed-methods model, the quantitative and qualitative data were first analyzed separately and then considered for an integrated analysis of the findings^40^. The quantitative data (including website use data) were analyzed using descriptive statistics (e.g., frequencies, percentages, means). For example, we provide a descriptive statistics summary (including mean, standard deviation, range, and median (25th quartile–75th quartile, Q1–Q3)) of the website use data (e.g., number of logins over 8 weeks for each participant). To evaluate the acceptability and safety of HoryzonsCa, we analyzed quantitative feedback from the HC-AUSI-Q and website use by calculating proportions (e.g., *n* (%) of participants who indicated they agreed or strongly agreed with specific items related to acceptability and safety; percentage of participants with at least 4 logins over the 8 weeks follow-up). To assess the pre-post outcomes of HoryzonsCa, a linear mixed model analysis with participant ID as random intercept was conducted on social functioning and clinical measures using restricted maximum likelihood estimation, producing an estimate of the pre-post change and its 95% confidence interval (CI) were reported for statistically significant changes between baseline and at 8 weeks follow-up. Before conducting this analysis, we conducted the checks of assumptions including testing the normality of residuals. We decided to use this analysis (instead of pre-post paired samples *t*-tests) to handle missing data (see Table 5), in consultation with a statistician. The linear mixed model analysis has the advantage of accounting for all available participant data, including participants with missing data, which can lead to higher statistical power with a small sample size. Multiple imputation prior to a mixed model approach has been shown to be not necessary as linear mixed models work well under the assumption of data that are missing at random, similarly to multiple imputation^41^. In addition, as an exploratory analysis, we used a mixed-effects logistic regression, with a random effect accounting for the correlation present within individuals, or McNemar’s test to compare the difference between baseline and 8 weeks follow-up in technology access, use, and competency (see Supplementary Table 1). In an exploratory analysis, we estimated correlations between website use and pre-post changes in outcomes within the total sample (Supplementary Table 3A) and within active users (Supplementary Table 3B). Quantitative data analysis was supported using SPSS (IBM Corp) or R [R Foundation for Statistical Computing]. For data preparation and analyses performed in R, we used the following R packages (but not limited to): the “dplyr,” “ggplot2,” “corr,” “survival,” and “lme4” packages. Two-sided *p* values <0.05 were considered statistically significant.

The qualitative data from the HC-AUSI-Q (i.e., short responses to open-ended questions) were entered into an Excel file (Microsoft Corporation), analyzed, and reviewed in relation to the concepts of acceptability, perceived benefits, safety, barriers, and suggestions for using HoryzonsCa. Two members of the research team reviewed all the qualitative data, co-developed a coding framework, and conducted a classical content analysis^42^ in consultation with the project lead. The coding framework was developed based on the research objectives, questions asked during the interview, and perspectives frequently emerging in the data. Patterns were identified based on topics being raised by at least four participants.

## Supplementary information

Supplementary Information

## Data Availability

All the data that support the findings of this study are not publicly available due to their containing information that could compromise the privacy of research participants; however, all the quantitative data from the survey are summarized in tables provided within the manuscript and the Supplementary Material.
