# Supplementary material for: Digital health innovation to prevent relapse and support recovery in young people with first-episode psychosis: A pilot study of Horyzons-Canada
Source: Schizophrenia (Heidelb). 2023 Apr 7;9(1):21. doi: 10.1038/s41537-023-00352-1 (PMC10082074; doi:10.1038/s41537-023-00352-1)
Supplement: Supplementary file 1 — Supplementary Information [file 41537_2023_352_MOESM1_ESM.docx]

**Digital health innovation to prevent relapse and support recovery in young people with first-episode psychosis: A pilot study of Horyzons-Canada**

Shalini Lal, BScOT, MSc, PhD; John F Gleeson, PhD; Simon D'Alfonso, BA, BSc, PhD; Hajin Lee, BSc, MA, PhD; Geraldine Etienne, MA; Ridha Joober, MD, PhD; Martin Lepage, PhD; Mario Alvarez-Jimenez, MAResearchMeth, DClinPsy, PhD

**SUPPLEMENTARY INFORMATION**

**Supplementary Table 1.** Technology Access and Use, and Competency Level (*N_baseline_* = 23, *N_follow-up_* = 19).

**Supplementary Table 2.** Qualitative feedback from the Horyzons-Canada Acceptability, Usability, Safety, and Impact Questionnaire (HC-AUSI-Q).

**Supplementary Table 3A.** Correlations between website usage and changes in pre-post outcomes among all participants (*N*_total_ = 20).

**Supplementary Table 3B.** Correlations between website usage and changes in pre-post outcomes among active participants (n = 13^*^). ^*^Participants who logged into the platform 4 times or more.

**Supplementary Table 1.** Technology Access and Use, and Competency Level (*N_baseline_* = 23, *N_follow-up_* = 19).

| **Question** | **Baseline** | | **8 Weeks Follow-Up** | | ***p*-value** |
| --- | --- | --- | --- | --- | --- |
|  | n | % | n | % |  |
| Do you have access to a smartphone? |  |  |  |  |  |
| Yes | 20 | 87 | 19 | 100 | 0.32^r^ |
| No | 3 | 13 | 0 | 0 |  |
| Type of Phone^a^ |  |  |  |  |  |
| iPhone | 13 | 57 | 13 | 68 | 0.96^s^ |
| Android | 7 | 30 | 6 | 32 |  |
| Do you have access to a Data plan?^b^ |  |  |  |  |  |
| Yes | 18 | 78 | 16 | 84 | 0.95^s^ |
| No | 4 | 17 | 3 | 16 |  |
| Data plan type^c^ |  |  |  |  |  |
| 0-6GB | 11 | 48 | 8 | 42 | 0.36^s^ |
| 7GB+ | 5 | 22 | 7 | 37 |  |
| Other (“HG”; “Koodo (Basic)”; “Roger”) | 2 | 9 | 1 | 5 |  |
| Do you have a computer at home? |  |  |  |  |  |
| Yes | 20 | 87 | 15 | 79 | 0.41^s^ |
| No | 3 | 13 | 4 | 21 |  |
| Do you have access to the internet? |  |  |  |  |  |
| Yes | 22 | 96 | 18 | 95 | 0.99^s^ |
| No | 1 | 4 | 1 | 5 |  |
| How often do you use the internet to search for mental health support?^d^ |  |  |  |  |  |
| More than once a week (Daily, 2-3 times per week)^e^ | 5 | 22 | 5 | 26 | 0.68^s^ |
| Once a week or less | 18 | 78 | 13 | 68 |  |
| Sites commonly used^f^ |  |  |  |  |  |
| Google | 11 | 48 | 6 | 32 |  |
| Institutional (ie, government, hospital, university) sites | 4 | 17 | 3 | 16 |  |
| YouTube | 4 | 17 | 2 | 11 |  |
| Social Media (ie, Facebook, Twitter, Instagram) | 1 | 4 | 2 | 11 |  |
| Forums/Blogs/Wikis | 4 | 17 | 1 | 5 |  |
| Horyzons | 0 | 0 | 4 | 21 |  |
| News sites/Journals | 0 | 0 | 2 | 11 |  |
| Other | 5 | 22 | 2 | 11 |  |
| Do you use social media to communicate with others? |  |  |  |  |  |
| Yes | 19 | 83 | 17 | 89 | 0.40^s^ |
| No | 4 | 17 | 2 | 11 |  |
| If yes, how often?^g^ |  |  |  |  |  |
| More than once a week (Daily, 2-3 times per week)^h^ | 18 | 78 | 16 | 84 | 0.94^s^ |
| Once a week or less | 1 | 4 | 1^i^ | 5 |  |
| Do you use text to communicate with others? |  |  |  |  |  |
| Yes | 20 | 87 | 19 | 100 | 0.32^r^ |
| No | 3 | 13 | 0 | 0 |  |
| If yes, how often?^j^ |  |  |  |  |  |
| More than once a week (Daily, 2-3 times per week)^k^ | 18 | 78 | 17 | 89 | 0.99^s^ |
| Once per week or less | 2 | 9 | 2 | 11 |  |
| Do you use email to communicate with others? |  |  |  |  |  |
| Yes | 16 | 70 | 14 | 74 | 0.82^s^ |
| No | 7 | 30 | 5 | 26 |  |
| If yes, how often? |  |  |  |  |  |
| More than once a week (Daily, 2-3 times per week)^l^ | 14^m^ | 61 | 9 | 47 | **0.008^s^** |
| Once per week or less | 2 | 9 | 5 | 26 |  |
| To what extent do you feel competent in using a computer? |  |  |  |  |  |
| Very or somewhat competent | 20 | 87 | 18 | 95 | 0.87^s^ |
| Neutral/somewhat not competent or Not competent^n^ | 3 | 13 | 1 | 5 |  |
| To what extent do you feel competent in navigating websites online? |  |  |  |  |  |
| Very or somewhat competent | 21 | 91 | 19 | 100 | NA^r^ |
| Neutral/somewhat not competent or Not competent^o^ | 2 | 9 | 0 | 0 |  |
| To what extent do you feel competent in using social media to communicate with others? |  |  |  |  |  |
| Very or somewhat competent | 21 | 91 | 19 | 100 | 0.32 ^r^ |
| Neutral/somewhat not competent or Not competent^p^ | 2 | 9 | 0 | 0 |  |
| To what extent do you feel competent in searching the Internet for mental health information, services and supports? |  |  |  |  |  |
| Very or somewhat competent | 19 | 83 | 16 | 84 | 0.54^s^ |
| Neutral/somewhat not competent or Not competent^q^ | 4 | 17 | 3 | 16 |  |

^a^20 participants (87%) who said yes to the question on whether they have access to a smartphone provided the specification for the type of phone at baseline.

^b^One participant (4%) did not provide data on having access to a Data plan at baseline.

^c^18 participants who said yes to the question on whether they have access to a data plan provided the specification for the type of data plan at baseline and 16 participants at the 8 weeks follow-up.

^d^One participant (5%) did not provide data on the use of the internet to search for mental health information, services, and supports at the 8 weeks follow-up.

^e^Two participants (9%) answered daily and 3 participants (13%) answered 2-3 times per week at baseline, and 1 participant (5%) answered daily and 4 participants (21%) answered 2-3 times a week at follow-up to the question on how often they use the internet to search for mental health support.

^f^More than one answer possible.

^g^19 participants who said yes to the question on whether they use social media to communicate with others provided data on the frequency for the use of social media at baseline and 17 participants at the 8 weeks follow-up.

^h^14 participants (61%) answered daily and 4 participants (17%) answered 2-3 times per week at baseline and 12 participants (63%) answered daily and 4 participants (21%) 2-3 times per week at follow-up to the question on how often they use social media to communicate with others.

^i^One participant (5%) was excluded from the 8 weeks follow-up, given that the participant answered no to the question on whether they use social media to communicate with others.

^j^20 participants (87%) who said yes to the question on whether they use text to communicate with others provided data on the frequency for the use of text at baseline.

^k^16 participants (70%) answered daily and 2 participants (9%) answered 2-3 times per week at baseline and 11 participants (58%) answered daily and 6 participants (32%) answered 2-3 times per week to the question on how often they use text to communicate with others.

^l^7 participants (30%) answered daily and 7 participants (30%) answered 2-3 times per week at baseline and 4 participants (21%) answered daily and 5 participants (26%) answered 2-3 times per week to the question on how often they use email to communicate with others.

^m^One participant (4%) was excluded from baseline, given that the participant answered no to the question on whether they use email to communicate with others.

^n^3 participants (13%) felt neutral in using a computer at baseline and 1 participant (5%) felt somewhat not competent or not competent in using a computer at follow-up.

^o^1 participant (4%) felt neutral and 1 participant (4%) felt somewhat not competent or not competent in navigating websites online at baseline.

^p^1 participant (4%) felt neutral and 1 participant (4%) felt somewhat not competent or not competent in using social media to communicate with others at baseline.

^q^2 participants (9%) felt neutral and 2 participants (9%) felt somewhat not competent or not competent in searching the Internet for mental health information, services and supports at baseline, and 2 participants (11%) felt neutral, and 1 participant (5%) felt somewhat not competent/not competent at follow-up.

^r^*P*-values are obtained using McNemar’s test, restricting to 19 participants with both baseline and follow-up assessments.

^s^*P*-values are obtained using mixed effects logistic regression, with a random effect accounting for the correlation present within individuals.

**Supplementary Table 2.** Qualitative feedback from the HC-AUSI-Q.

| **Topic** | **Themes** | **Sample Quotes**^a^ | **n** |
| --- | --- | --- | --- |
| **Acceptability of the Horyzons Platform** | | | |
| **Helpfulness^c^** | User interactions on the platform (with other users; with moderators) | *Not enough sharing. Didn't bring anything extra in my evolution^b^.* [Participant 7]  *Valerie [clinician]: she called me on my cell phone. She's very nice, She talked half an hour to me. She wanted to make sure I was safe.* [Participant 9] | 10 |
|  | Low user activity and interactions on the platform | *I didn't really talk to people, went to the chat but did not do much.*  [Participant 16]  *There was not a lot of people. Nobody online, I did not have a chance to interact.* [Participant 14] | 8 |
| **Ease of Use^d^** | Clarity of content | *Even to an elementary child - the website is easy to understand.* [Participant 5]  *Well written out. Not make it difficult. Everything was described properly.* [Participant 10] | 10 |
|  | Content presentation (e.g., comics, meditation audio files, help resources) | *Because of the cartoons.* [Participant 5]  *I like the little photo like comic book. I like the Australian accent meditation.* [Participant 14] | 6 |
|  | Navigation | *It was really easy to surf on the website.* [Participant 12] | 3 |
| **Obstacles^e^** | System Obstacles |  |  |
|  | Technical (e.g., remembering password, mobile applications, moderation of the platform) | *Login to use (another username + password to remember.* [Participant 15]  *Would be better if it was an app, give you more opportunities if you were travelling.* [Participant 17] | 9 |
|  | Content (e.g., the topics addressed) and content presentation | *Finding reading boring (since the website's principal activity is reading).* [Participant 1]  *Hard to relate to the content, mostly for teenagers (had to put examples to my own context).* [Participant 6]  *Too much info. We get lost.* [Participant 12]  *I don't find [that] with the cartoon pictures it helps much.* [Participant 8] | 8 |
|  | Personal External Barriers (e.g., lack of time or willingness to use the platform, personal traits/preferences, interpersonal barrier) | *Not having enough time.* [Participant 10]  *Not being sure that who you're interacting with (you're not sure who they are). They could [be] apprehensive.* [Participant 20] | 7 |
| **Improvement Suggestions^f^** | System features (e.g., moderators, mobile applications, symptom tracker, frequent updates, monthly summary, design improvements, automatic sign in, websites categorized by age, connecting deeper with other users) | *If they start with positive effects. Weekly updates of what's happening in the café.* [Participant 6]  *Keep a journal so that they (young people) know how they're feeling (they keep track of everything).* [Participant 10]  *An app. Progress meter: something that would track how you feel.* [Participant 15] | 12 |
|  | Content (e.g., inclusion of more engaging components such as video, video games, etc.) | *Make the website a sort of game. Have interesting contents have little games.* [Participant 1]  *There should be one task per day on a particular strength^b^.* [Participant 7]  *Maybe visual component more video than are necessary related to the audio.* [Participant 17] | 10 |
|  | Inter-user interaction on the platform (e.g., more users on the platform, encourage interactions between users, more group activities) | *Participants get the possibility to post about their problems and how they're feeling this week/today.* [Participant 9]  *A group activities.* [Participant 12]  *More people for more interactions.* [Participant 13]  *When I posted something - wanted other people to answer, not only the moderator.* [Participant 14] | 8 |
|  | Platform promotion (e.g., use of social media, media, word of mouth, advertisements, website demonstration) | *In general, you could show the website and explain to them how it works.* [Participant 9]  *Insite all people who are at PEPP to talk about HORYZONS.* [Participant 13] | 6 |
|  | Personal engagement with the platform | *Focus on the positive effects. Keep trying.* [Participant 6]  *They (young people) should choose like a time to go on it everyday.* [Participant 10] | 4 |
| **Platform Promotion^g^** | Videos | *Most effective method is videos.* [Participant 5] | 8 |
|  | Media promotion (e.g., talk shows, TV ads, web ads, social media, school website) | *Info websites (one page website).* [Participant 6]  *Social media.* [Participant 10] | 8 |
|  | Demonstrations / Reference by professionals or peers (e.g., testimonies) | *A group at a certain date where you talk about the website. A mention it is safe and confidential.* [Participant 9]  *Individual meeting: show the website each section of the website.* [Participant 13]  *Life reviews: how the website changed people (testimonies).* [Participant 5] | 8 |
|  | Pamphlets | *Pamphlet that explains the website (a powerpoint too) so they can look and say ok! Ok! This is interesting.* [Participant 9] | 3 |
| **Safety of the Horyzons platform** | | | |
| **Safety^h^** | **User Safety** |  |  |
|  | Safe Space (understanding between users) | *I didn't think that any bullying activities could happen on this website.* [Participant 1]  *Everyone there has a reason to be there. They're not judging you, its confidential.* [Participant 10] | 9 |
|  | Trust in clinician team’s credibility, understanding, and professionalism | *Cause they were a team of professionals.* [Participant 3]  *Maybe because it was recommended by the hospital.* [Participant 16] | 6 |
|  | Limited concerns about safety | *It's just a website - did not feel the need to be protected.* [Participant 13] | 4 |
|  | **System Safety** |  |  |
|  | System security (e.g., passwords, no advertisements, anonymity, privacy of the posts) | *Anonymous platform. Safe: the information is not posted somewhere else.* [Participant 6]  *There were no ads pop up. No advertisements.* [Participant 18]  *You have to sign in to get access to the info.* [Participant 20] | 7 |
|  | Safe content (e.g., credible resources, provided helpful list of crisis resources) | *Just because of the info and the credible resources.* [Participant 20]  *If there's an urgency, we have contacts luke 'l'autre maison”.* [Participant 9] | 3 |

Abbreviations: HC-AUSI-Q, Horyzons-Canada Acceptability, Usability, Safety, and Impact Questionnaire; PEPP, Prevention and Early Intervention Program for Psychosis; n = Frequency.

^a^Some of the open-ended responses provided by a participant were split into multiple comments and then analysed.

^b^We translated participants’ comments (originally written in French) into English, first by a fluent English speaker (HL) using DeepL, followed by validation by a native French speaker (lab member), and final validation by the project lead (SL) who is a native English speaker, fluent in French.

^c^Of the 20 participants, **18 individuals** responded to the open-ended question that asked whether other Horyzons-Canada users helped their recovery on Horyzons-Canada; ^d^**17 individuals** responded to the open-ended question that asked whether the information on Horyzons-Canada was easy to understand; ^e^**20 individuals** responded to the open-ended question that asked to list 3 key obstacles that might prevent a young person from using Horyzons-Canada; ^f^**19 individuals** responded to the open-ended question that asked to list 3 strategies that would facilitate/encourage a young person to use Horyzons-Canada; **19 individuals** responded to the open-ended question that asked to list 3 strategies that would encourage a young person to use Horyzons-Canada for more than 3 months to 1 year; **19 individuals** responded to the open-ended question that asked what could be added to Horyzons-Canada to improve it for future users; ^g^**18 individuals** responded to the open-ended question that asked how best to introduce youth to Horyzons-Canada for the first time (i.e. open demonstration session, self-exploration, pamphlets, videos, peer-orientation, etc.); ^h^**19 individuals** responded to the open-ended question that asked whether they felt safe on Horyzons-Canada; **16 individuals** responded to the open-ended question that asked whether they felt like the information shared on Horyzons-Canada was confidential.

**Supplementary Table 3**

1. Correlations between website usage and changes in pre-post outcomes among all participants (*N*_total_ = 20)

| Measure | Logins^a^ | Newsfeed posts^b^ | Newsfeed comments^c^ | Pathways^d^ | Steps^e^ | Actions^f^ | Talk-it-outs^g^ |
| --- | --- | --- | --- | --- | --- | --- | --- |
| SOFAS, n=19 | 0.43 | 0.27 | 0.41 | 0.19 | 0.29 | 0.28 | 0.16 |
| PSP, n=18 | 0.36 | 0.29 | 0.26 | -0.17 | 0.01 | -0.07 | 0.26 |
| CGI, n=14 | -0.47 | -0.14 | -0.41 | -0.36 | **-0.57^*^** | **-0.59^*^** | -0.20 |
| Global SANS (excluding attention), n =19 | -0.45 | -0.03 | -0.28 | -0.30 | -0.25 | -0.42 | -0.20 |
| BPRS, n=19 | -0.05 | 0.09 | 0.04 | 0.06 | -0.09 | -0.08 | -0.02 |
| MSPSS | 0.17 | -0.13 | 0.10 | 0.00 | 0.01 | 0.06 | 0.14 |
| CDS, n=19 | -0.19 | 0.03 | -0.08 | 0.07 | -0.14 | -0.08 | -0.08 |
| Global SAPS, n=19 | -0.28 | -0.13 | -0.17 | -0.13 | -0.31 | -0.12 | -0.32 |
| SES | 0.29 | 0.04 | 0.16 | 0.08 | 0.18 | 0.15 | 0.25 |
| SKS | 0.16 | -0.23 | 0.06 | 0.29 | 0.30 | 0.10 | 0.07 |
| SUS | 0.13 | -0.26 | 0.01 | 0.13 | 0.15 | 0.09 | 0.12 |

1. Correlations between website usage and changes in pre-post outcomes among active participants (n = 13)^h^

| Measure | Logins^a^ | Newsfeed posts^b^ | Newsfeed comments^c^ | Pathways^d^ | Steps^e^ | Actions^f^ | Talk-it-outs^g^ |
| --- | --- | --- | --- | --- | --- | --- | --- |
| SOFAS, n=12 | 0.47 | 0.31 | 0.48 | 0.18 | 0.27 | 0.39 | 0.13 |
| PSP, n=12 | 0.36 | 0.32 | 0.22 | -0.34 | -0.15 | -0.14 | 0.27 |
| CGI, n=9 | -0.26 | -0.05 | -0.30 | -0.27 | -0.45 | -0.55 | -0.07 |
| Global SANS (excluding attention), n=12 | -0.32 | 0.15 | -0.17 | -0.31 | -0.05 | -0.52 | -0.12 |
| BPRS, n=12 | 0.30 | 0.34 | 0.31 | 0.26 | 0.03 | -0.09 | 0.08 |
| MSPSS | 0.37 | -0.10 | 0.20 | 0.04 | 0.10 | 0.11 | 0.20 |
| CDS, n=12 | 0.13 | 0.25 | 0.15 | 0.34 | 0.07 | -0.06 | 0.06 |
| Global SAPS, n=12 | 0.14 | 0.00 | 0.06 | 0.00 | -0.11 | 0.10 | -0.33 |
| SES | 0.31 | -0.01 | 0.15 | 0.05 | 0.16 | 0.14 | 0.31 |
| SKS | 0.23 | -0.33 | 0.06 | 0.36 | 0.44 | 0.20 | 0.08 |
| SUS | 0.32 | -0.23 | 0.10 | 0.19 | 0.28 | 0.18 | 0.18 |

Abbreviations: SOFAS, Social and Occupational Functioning Assessment Scale; PSP, Personal and Social Performance Scale; CGI, Clinical Global Impression; SANS, Scale for the Assessment of Negative Symptoms; BPRS, Brief Psychiatric Rating Scale; MSPSS, Multidimensional Scale of Perceived Social Support; CDS, Calgary Depression Scale; SAPS, Scale for the Assessment of Positive Symptoms; SES, Self-Esteem Rating Scale; SKS, Strengths Knowledge Scale; SUS, Strengths Use Scale.

^a^Number of logins over 8 weeks.

^b^Number of newsfeed posts made by participants in the peer-to-peer web-based social networking (the Café).

^c^Number of newsfeed comments made by participants in the peer-to-peer web-based social networking (the Café).

^d^Number of Pathways that participants completed as part of engagement with therapeutic content.

^e^Number of Steps (each “Pathway” comprised of a series of related "Steps”) that participants completed as part of engagement with therapeutic content.

^f^Number of Actions (activities designed to reinforce strengths or practice new skills) that participants completed as part of engagement with therapeutic content.

^g^Number of Talk-it-outs posts and comments in which participants discuss specific issues (e.g., handling setbacks) and receive support or suggestions.

^h^Participants who logged into the platform 4 times or more. We chose the cutoff value of 4 based on our definition of minimum platform usage as an average of at least one login per 2 weeks (4 total logins) during the period of an 8-week intervention.

^*^*p* < .05 (two-tailed).
